# Unsatisfactory response to acute medications does not affect the medication overuse headache development in pediatric chronic migraine

**DOI:** 10.1186/s10194-024-01766-7

**Published:** 2024-04-23

**Authors:** Ilaria Frattale, Michela Ada Noris Ferilli, Fabiana Ursitti, Giorgia Sforza, Gabriele Monte, Martina Proietti Checchi, Samuela Tarantino, Luigi Mazzone, Massimiliano Valeriani, Laura Papetti

**Affiliations:** 1 Child Neurology and Psychiatry Unit, Department of Wellbeing of Mental and Neurological, Dental and Sensory Organ Health, Policlinico Tor Vergata Foundation Hospital, Rome, Italy; 2 Developmental Neurology, Bambino Gesù Children’ s Hospital, IRCCS, Piazza di Sant’Onofrio 4, 00165 Rome, Italy; 3 System Medicine Department, Tor Vergata University of Rome, Rome, Italy; 4 Center for Sensory-Motor Interaction, Aalborg University, Aalborg, Denmark

**Keywords:** Childhood, Adolescents, Chronic migraine, Medication overuse headache, Acute treatment

## Abstract

**Background:**

Chronic migraine (CM) negatively impacts the quality of life of 2 to 4% of pediatric patients. In adults, CM is frequently linked to medication overuse headache (MOH), but there is a much lower prevalence of MOH in children. A suboptimal response to acute therapies may lead to their reduced use, thus preventing MOH development in children and adolescents. The frequency of patients with CM who do not respond to acute therapies was examined in the present study. We investigated whether the prevalence of MOH was different between responders and non-responders. We also examined whether patients receiving prophylactic therapy had an improved response to acute therapy. Finally, we investigated if there was a difference in the frequency of psychiatric comorbidities between responders and non-responders.

**Methods:**

We retrospectively analysed clinical data of all chronic pediatric migraineurs under the age of 18 referred to the Headache Centre at Bambino Gesù Children Hospital in June 2021 and February 2023. ICHD3 criteria were used to diagnose CM and MOH. We collected demographic data, including the age at onset of migraine and the age of the CM course. At baseline and after 3 months of preventive treatment, we evaluated the response to acute medications. Neuropsychiatric comorbidities were referred by the children’s parents during the first attendance evaluation.

**Results:**

Seventy patients with CM were assessed during the chosen period. Paracetamol was tried by 41 patients (58.5%), NSAIDs by 56 patients (80.0%), and triptans by 1 patient (1.4%). Fifty-one participants (73%) were non-responder to the abortive treatment. The presence of MOH was detected in 27.1% of the whole populations. Regarding our primary aim, MOH was diagnosed in 29% of non-responder patients and 22% of responders (*p* > 0.05). All patients received preventative treatment. After 3 months of preventive pharmacological therapy, 65.4% of patients who did not respond to acute medications achieved a response, while 34.6% of patients who were non-responder remain non-responder (*p* < 0.05). Prophylactic therapy was also effective in 69% of patients who responded to acute medication (*p* < 0.05). Psychiatric comorbidities were detected in 68.6% of patients, with no difference between responders and non-responders (72.2% vs. 67.3%; *p* = 0.05).

**Conclusions:**

Despite the high prevalence of unresponsiveness to acute therapies in pediatric CM, it does not act as a protective factor for MOH. Moreover, responsiveness to acute drugs is improved by pharmacological preventive treatment and it is not affected by concomitant psychiatric comorbidities.

## Introduction

Migraine is the primary cause of pain in childhood and its frequency increases with age, overtaking 20% in adolescence [1]. Throughout life, the frequency, severity, and disability of this condition can fluctuate. The transition between episodic and chronic forms is possible [2–5], and the origin of this modification is not fully understood [6].

Around 2 to 4% of children with migraines experience chronic migraine (CM) [7–9]. According to the ICHD3 criteria, CM is defined by the presence of at least 15 headache days for a minimum of 3 months with at least 8 migraine days each month [10]. Chronic headaches can negatively impact one’s quality of life and the ability to complete routine school and sports activities, as well as maintain social relationships [11].

Central sensitization changes can be caused by the increased use of painkillers, which can decrease the effectiveness of acute therapies [12–15]. This phenomenon may be responsible of frequent acute medication intakes, MOH development, and migraine chronification [6, 16–18].

The definition of response to acute treatment, which encompasses NSAIDs, triptans, and combinations, is pain relief within 2 h and a state of well-being that lasts for at least 24 h [19]. Oral formulation is the most used by migraineurs with a 2-hour pain free rate from 19 to 40% [20, 21], while the subcutaneous administration (e.g. triptans) leads to pain free between 60 and 65% of cases [22]. Factors like late intake, inadequate dosage, and formulations with inadequate absorption can lead to a failed response to treatment [23]. During the chronic course of migraine, acute therapy response is observed to be lower. The concomitant conditions of obesity and neuropsychiatric comorbidities can cause resistance to acute medications [24].

According to pediatric studies, MOH is less prevalent in children and adolescents than in adult patients [25–29]. Although the reason is not known, factors related to brain development and the progression of migraine could explain the different prevalence of MOH in pediatric migraines and adulthood [28]. It can be inferred that pediatric patients with CM may not always respond to pharmacological treatments for acute therapy, which could prevent them from taking analgesic drugs frequently. If this were true, we would expect a lower prevalence of MOH in patients who do not respond than in those who respond to acute therapy.

The primary aims of our study were: (1) to calculate the frequency of pediatric patients with CM who exhibited resistance to the acute therapies, and (2) to investigate whether MOH prevalence was different between responders and non-responders to the analgesic drugs. As secondary aims, we examined: (1) the impact of prophylactic treatment on the response to acute treatment and (2) the different frequency of psychiatric comorbidities between responders and non-responders to acute therapy.

## Methods

### Patients

We retrospectively analyzed clinical data of all patients affected by CM who attended the Headache Centre at Bambino Gesù Children’s Hospital between June 2021 and February 2023. Data was obtained from patients between 6 and 17 during the initial visit and a second follow-up visit after three months. We used ICHD3 criteria [10] to diagnose CM and MOH.

We analyzed demographic characteristics, including sex, age at onset of migraine, age at CM onset, frequency of the attacks, MOH diagnosis, and response to acute medications at baseline and after 3 months of preventive treatment. Neuropsychiatric comorbidities were referred by the children’s parents during the first attendance evaluation.

Paracetamol, nonsteroidal anti-inflammatory drugs (NSAIDs), and triptans were considered as acute medication in their own category. A patient was defined as not responding to acute therapy when he did not respond to paracetamol, an NSAID, and a triptan. Also, for the definition of non-responder, we verified the correct use of acute therapy in terms of adequate dosage and adherence. We assumed that due to local legislative reasons and the age of patients, the prescription of triptans was not common. For this reason, we also considered those who had no response to paracetamol and two NSAIDs as not responders.

We only included subjects with CM for whom we have data on the use of acute therapies (dosage, class of drug) at baseline and after three months of prophylactic treatment.

### Statistical analysis

Statistical analysis was conducted using SPPS version 22.0 and consisted of three steps.

The initial step involved a descriptive analysis that reported the differences between the responders and non-responders to analgesic drugs. In particular, for the categorical variables (sex, response or not to prophylactic therapy, and the presence or absence of psychiatric comorbidities) we considered the χ2 test, while for the ordinary variables (age) we used the analysis of variance (ANOVA).

The second step was a bivariate analysis to study how each variable, considered individually, correlated with either response or resistance to acute therapy. Different statistical tests were used depending on the nature of the covariates and the response. For ordinal categorical variables, we used the Mann-Whitney U-test, the Kruskal-Wallis test and the Spearman’s Rho. For the numeric variables, we transformed the response using the log scale ratio and considered the t-test, ANOVA, and Spearman’s Rho. The individual tests in the bivariate analysis did not consider the contemporary effect of other covariates.

The third step was a multivariate analysis where all the variables that in the bivariate analysis showed a p-value ≤ 0.2 in determining response or resistance to acute therapy were included. A generalized linear model (GLM) with cumulative link and proportional odds assumption was utilized for multivariate analysis. A p-value being ≤ 0.05 was deemed significant.

The parents of the participants provided written informed consent. The study was approved by the Ethics Committee of Bambino Gesù Children Hospital.

## Results

In the considered period of time considered, 1680 children and adolescents were visited at our Headache Center. According to ICHD 3 criteria, 81 patients (4.8%) were diagnosed with CM. Eleven patients were excluded from further analysis since we did not have enough data on the use of acute therapy (untried or assumed at inadequate dosages). The analysed population included 70 patients (58 females and 12 males) with an average age at the time of the first visit of 14.2 ± 2.4 years. Demographic features of the sample are summarized in Table 1.

**Table 1:** Demographic Characteristics of n=70 patients in the analysed period

Patients with follow up:	70
Female: male	58:12 (females 83% - males 17%)
Mean age at the visit	14.2 ± 2.4
Mean age at migraine onset	11.2 ± 3.2
Mean age at chronification onset	13.6 ± 2.3
High- frequency migraine before chronification (%)	n = 61 (87%)
Preventative treatment before chronification (%)	n = 20 (28.5%)
Acute Medication tried before (%)	Paracetamol n = 41 (58.6%) NSAIDs n = 56 (80%) Triptan n = 1 (1.4%)
Responder vs. Non Responder Baseline (%)	n = 19/70 vs. n = 51/70 (27% vs. 73%)
Responder vs. Non Responder After preventive (%)	n = 41/58 vs. n = 17/58 (71.4% vs. 28.6%)
MOH MOH in Responder vs. Non Responder %	n = 19/70 (27.1%) 22% vs. 27%
Neuropsychiatric comorbidities Neuropsychiatric comorbidities in Responder vs. Non Responder %	n = 48/70 (69%) 72.2% vs. 67.3%

The average age of migraine onset was 11.2 ± 3.2 years, while the average age of CM onset was 13.6 ± 2.3 years. Migraine lasted an average of 2.4 years from its onset before becoming chronic. Before having CM, 87% of patients had a high-frequency episodic migraine (EM) (more than 5 attacks per month). In 28.5% of cases, a prophylactic treatment had been tried and resulted ineffective.

Before arriving at our center, 41 out of 70 patients had tried paracetamol (58.6%), 56 NSAIDs (80%), and only 1 triptans (1.4%).

**Fig. 1:**
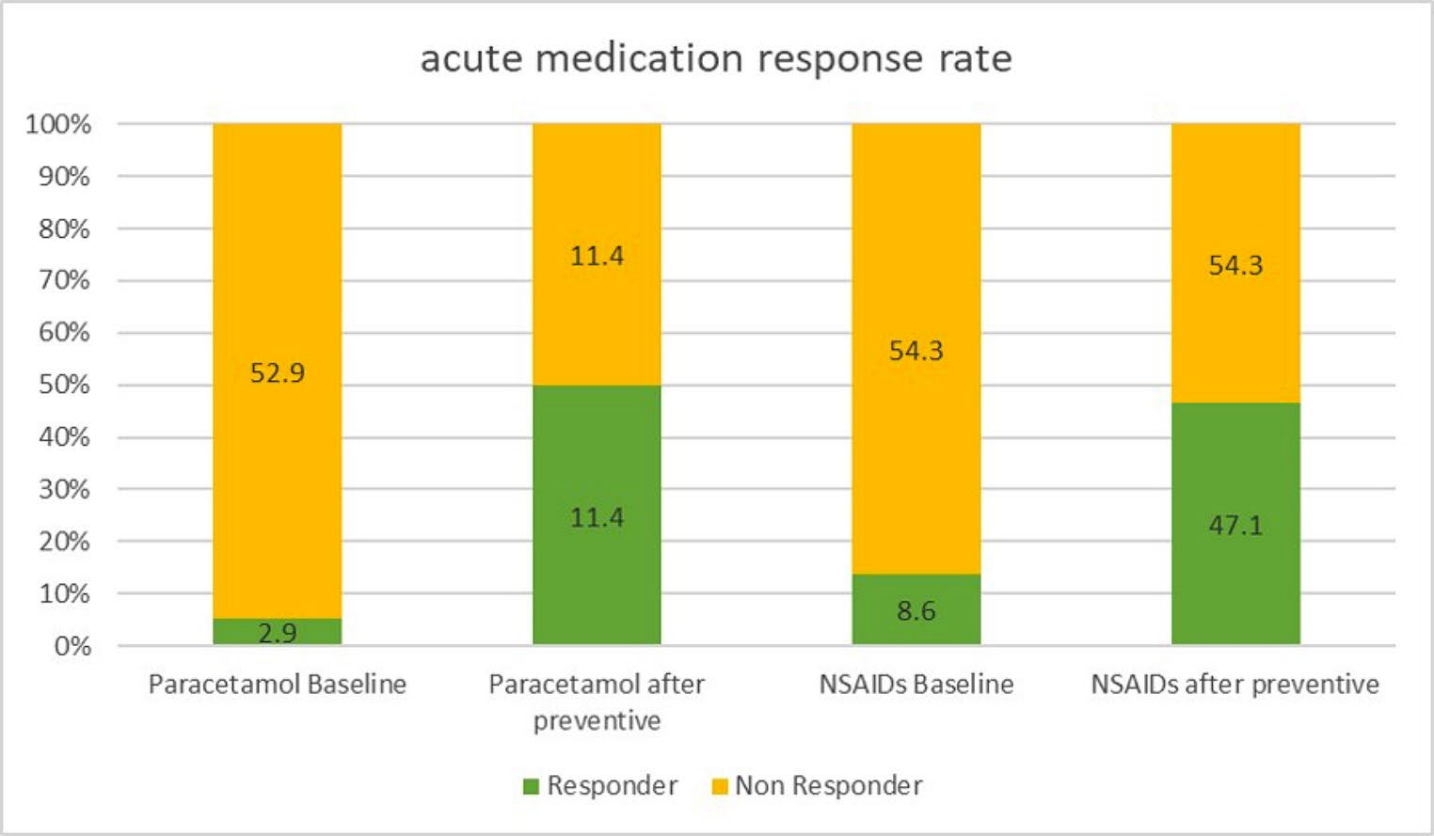
Acute medications response rate at baseline and after 3-months-preventative treatment

Fifty-one participants (73%) were non-responder to the acute treatment, while 19 (27%) were responsive. Response to acute treatment was independent of the drug (Fig. 1).

Response or resistance to the acute therapy did not depend on the age at migraine onset (mean age: 11.2 vs. 11.4 years; *p* > 0.05) and at CM onset (mean age: 13.8 vs. 13.5 years; *p* > 0.05), and the duration of the disease (32.3 vs. 27.5 months; *p* > 0.05).

Among the total subjects (*n* = 70), in 19 (27.1%) of cases, there was a concomitant presence of MOH. In these patients, an average of 23.5 doses of acute medications per month were assumed. MOH prevalence was similar among non-responders and responders (29% vs. 22%; *p* > 0.05).

All patients were given prophylactic pharmacological therapy, and the response was confirmed at a follow-up visit three months after the start. Twelve patients did not assume the prescribed therapy or suspended it too early (within a month). A decrease in headache days per month ≥ 50% was observed in 58% (34/58) of patients who received prophylaxis. After prophylaxis, the prevalence of patients responding to the analgesic drugs increased to 71.4% (41/58), while non-responder patients decreased to 28.6% (17/58). More in detail, 65.4% of subjects who failed to respond to acute therapy before initiating prophylaxis achieved a response after 3 months of preventive pharmacological therapy. The remaining non-responder patients (34.6%) kept not responding to acute therapy (*p* < 0.05). Among the patients who responded to the acute therapy, 69% simultaneously responded to the prophylactic therapy, while 31% did not experience a significant change in headache days per month (*p* < 0.01). The bivariate analysis identified only prophylactic therapy as a factor associated with response to acute therapy (R 0.9; C.I. 0.7–0.95; *p* < 0.05).

Psychiatric comorbidities were referred in 48 of patients (69%). Anxiety and depressive disorders were the most prevalent psychiatric conditions. The frequency of psychiatric comorbidities did not differ significantly between responders and non-responders to analgesic drugs (72.2% vs. 67.3%; *p* = 0.05).

## Discussion

In both pediatric and adult migraine patients, resistance to acute therapy is a significant issue. In this context, our study in a pediatric cohort of CM patients produced the following significant findings:

1. Most CM patients found acute medications to be ineffective.
2. Both responders and non-responders had the same MOH prevalence, indicating that MOH development is not affected by failure to abort medication.
3. After receiving preventive treatment for three months, most patients, particularly those with good outcomes after prophylaxis, returned to respond to acute medication.
4. The response to acute medication was not influenced by psychiatric comorbidities.

### Poor response to acute therapy in children and adolescents with CM

Acute medications were found to be ineffective for the majority of CM patients. The results confirm our hypothesis that CM patients have a decreased response to acute therapy. Whether poor response to acute therapy is a risk factor for the development of CM or merely a consequence of migraine severity still remains a debated question [6]. In patients with episodic migraine, suboptimal response to acute treatment was associated with a risk of developing CM within one year, independently of other migraine features, such as disability and frequency of the attacks [23]. Acute treatment failure can result in more frequent and longer attacks, as well as greater disability, which can lead to the onset of CM [6, 12–15]. In this view, more effective acute treatments should decrease the chances of developing CM. Multiple treatments are often necessary to overcome resistance to acute drugs in CM patients [23]. Our findings, showing that non-responders are resistant to at least two categories of drugs, including paracetamol, NSAIDs, and triptans, agree with previous results. The main reason for the resistance to analgesic drugs in CM is the severity of the disease, as evidenced by the weak response to multiple acute therapies [6]. Migraine progression leads to central sensitization, which in turn could reduce the response to acute therapies [6]. Poor response to acute therapy and the overuse of acute medications both contribute to migraine progression and the onset of MOH [16–18, 23, 30, 31].

### Might poor response to acute therapy reduce the risk of MOH?

A primary aim of this study was to investigate whether the poor response to analgesic drugs could contribute to the lower prevalence of MOH in children and adolescents, as compared to adults [25]. Indeed, patients with suboptimal response to acute therapy may be discouraged from using it frequently. Compared to the adult population with chronic headache in which MOH has a prevalence of 64% [32], in pediatric age MOH is far rarer, ranging from 20 to 50% of chronic patients [27–29]. Since the possibility of MOH development raises with increasing age, the parental control over drug intake could partly explain the difference between children and adulthood [33]. MOH pathophysiology is very complex and not fully understood. Beyond the amount of analgesic drug intake, other factors, such as medication effects [34], genetics factors [35] and headache-specific pain pathways [36], are involved.

In our sample, MOH was diagnosed in 25% of patients with a similar prevalence in both responders and non-responders. This result did not confirm our initial hypothesis of a key role played by the poor response of analgesic drugs in explaining the rather low prevalence of MOH in children compared to adulthood. Therefore, we can conclude that in pediatric age the unresponsiveness to the acute therapy does not have a protective role for MOH development.

One may wonder why non-responders keep assuming drugs which, however, do not help them in reducing pain. Though not completely known, this behavior suggests the possibility of an addictive psychopathological profiles of either the patient or her/his parents who administer the drug [37, 38].

### Are prophylactic therapies useful in pediatric migraine?

Our study revealed that more than 70% of patients who received prophylactic therapy experienced a significant improvement in their response to acute medication. The number of headache days per month decreased by more than 50% for 69% of them, compared to the three months before treatment.

In children and adolescents, the use of pharmacological prophylactic treatment is not as widely accepted as for adults. In 2014, topiramate was approved by the Food and Drug Administration for the prophylactic treatment of migraine in children over 12 years based on robust clinical results [39, 40]. Unfortunately, there are only limited data for other drugs which are not licenced for pediatric age [33, 41–45]. Monoclonal antibodies and gepants are still subject to clinical trials and cannot be prescribed. Second, the usefulness of the prophylactic pharmacological treatments in children and adolescents has been challenged by the CHAMP study, which failed in showing a superiority of either topiramate or amitriptyline over placebo [41]. Powers et al. recommended that psychological treatments should be the preferred treatment over medication because of the possible side effects and high placebo efficacy rate.

Although our study was not designed to investigate the efficacy of pharmacological prophylactic treatments and we did not consider a control group with placebo, more than half of our patients undergone prophylaxis showed a significant reduction in headache days per month. However, what is most noteworthy within the present results is that more than two third of our patients improved their response to analgesic drugs after prophylaxis. Though needing confirmation in appropriately designed trial, our present findings suggest that the response to acute therapy should be considered in future clinical studies on migraine prophylaxis in children and adolescents [41].

Our study was not designed to investigate the efficacy of pharmacological prophylactic treatments in pediatric CM. Despite this and the absence of a control group with placebo, we found that more than half of our patients undergone prophylaxis showed a significant reduction in headache days per month. Our findings indicate that over two-thirds of our patients have improved their ability to respond to acute therapies after prophylaxis. Though needing confirmation in an appropriately designed trial, we suggest that the response to acute therapy should be considered in future clinical studies on migraine prophylaxis in children and adolescents.

### The effect of psychiatric comorbidities on the response to acute therapy

Anxiety and depression are highly prevalent in children and adolescents with migraines [46, 47]. The presence of these disorders can predict a poor response to acute and preventive therapy and a greater disability [48, 49]. On the other hand, headache chronification may lead to reduced quality of life and disability, which can be represented as factors for the onset of mood disorders [50]. From this perspective, it is possible that neuropsychiatric disturbances could play a role in determining the response to acute therapy.

In our study, almost 70% of patients had psychiatric comorbidities, which caused the same poor response to analgesic drugs as those without psychiatric symptoms. In conclusion, our data suggest that in pediatric patients with CM, the response to analgesic drug is not affected by neuropsychiatric comorbidities.

## Limitations

The small number of patients and retrospective nature of our study result in some limitations. The retrospective design of the study increases the risk of recall bias and incomplete data. Since most patients came to our attention already showing a CM, we do not have detailed information about the previous migraine time course. Furthermore, we lack knowledge about the response to acute therapy for most patients prior to migraine chronification. It’s impossible to determine with certainty whether the poor response to acute medications is caused by chronification mechanisms or if it’s not dependent on them. Finally, in our patients, neuropsychiatric comorbidities were diagnosed based on what was referred by the patients and their parents. Validated tools for diagnosing psychiatric disturbances are necessary to determine their impact on the response to acute medications.

Further longitudinal studies would provide more reliable evidence on the relationship between acute therapy response and MOH development.

## Conclusion

In conclusion, our results suggest that the poor response to analgesic drugs observed in our patients with CM does not explain the rather low prevalence of MOH. Furthermore, we discovered that using a preventative treatment may enhance the therapeutic response to acute medication, thus reducing the disability related to the migraine attack. Lastly, although psychiatric comorbidities should be considered in the whole assessment of CM patients, they do not affect the response to acute therapy.

## Data Availability

The data that support the findings of this study are not openly available due to reasons of sensitivity and are available from the corresponding author upon reasonable request. Data are located in controlled access data storage at Bambino Ges? Children?s Hospital, Rome.

## Declarations

### Clinical trial number

not available.
